# Pharmacogenetic stimulation of cholinergic pedunculopontine neurons reverses motor deficits in a rat model of Parkinson’s disease

**DOI:** 10.1186/s13024-015-0044-5

**Published:** 2015-09-23

**Authors:** Ilse S. Pienaar, Sarah E. Gartside, Puneet Sharma, Vincenzo De Paola, Sabine Gretenkord, Dominic Withers, Joanna L. Elson, David T. Dexter

**Affiliations:** Centre for Neuroinflammation and Neurodegeneration, Division of Brain Sciences, Faculty of Medicine, Imperial College London, London, W12 ONN UK; Department of Applied Sciences, Faculty of Health and Life Sciences, Northumbria University, Newcastle upon Tyne, NE1 8ST UK; Institute of Neuroscience, Newcastle University, Framlington Place, Newcastle upon Tyne, NE2 4HH UK; Medical Research Council Clinical Sciences Centre, Faculty of Medicine, Imperial College London, London, W12 0NN UK; Institute of Genetic Medicine, Newcastle University, Newcastle upon Tyne, NE1 3BZ UK; Centre for Human Metabonomics, North-West University, Potchefstroom, South Africa

**Keywords:** Cholinergic, Deep brain stimulation, DREADD, Parkinson’s disease, Pedunculopontine nucleus

## Abstract

**Background:**

Patients with advanced Parkinson's disease (PD) often present with axial symptoms, including postural- and gait difficulties that respond poorly to dopaminergic agents. Although deep brain stimulation (DBS) of a highly heterogeneous brain structure, the pedunculopontine nucleus (PPN), improves such symptoms, the underlying neuronal substrate responsible for the clinical benefits remains largely unknown, thus hampering optimization of DBS interventions. Choline acetyltransferase (ChAT)::Cre^+^ transgenic rats were sham-lesioned or rendered parkinsonian through intranigral, unihemispheric stereotaxic administration of the ubiquitin-proteasomal system inhibitor, lactacystin, combined with designer receptors exclusively activated by designer drugs (DREADD), to activate the cholinergic neurons of the nucleus tegmenti pedunculopontine (PPTg), the rat equivalent of the human PPN. We have previously shown that the lactacystin rat model accurately reflects aspects of PD, including a partial loss of PPTg cholinergic neurons, similar to what is seen in the post-mortem brains of advanced PD patients.

**Results:**

In this manuscript, we show that transient activation of the remaining PPTg cholinergic neurons in the lactacystin rat model of PD, via peripheral administration of the cognate DREADD ligand, clozapine-N-oxide (CNO), dramatically improved motor symptoms, as was assessed by behavioral tests that measured postural instability, gait, sensorimotor integration, forelimb akinesia and general motor activity. *In vivo* electrophysiological recordings revealed increased spiking activity of PPTg putative cholinergic neurons during CNO-induced activation. c-Fos expression in DREADD overexpressed ChAT-immunopositive (ChAT+) neurons of the PPTg was also increased by CNO administration, consistent with upregulated neuronal activation in this defined neuronal population.

**Conclusions:**

Overall, these findings provide evidence that functional modulation of PPN cholinergic neurons alleviates parkinsonian motor symptoms.

**Electronic supplementary material:**

The online version of this article (doi:10.1186/s13024-015-0044-5) contains supplementary material, which is available to authorized users.

## Background

The pedunculopontine nucleus (PPN), located in the dorsal tegmentum of the midbrain and upper pons, regulates aspects of cognition, sleep architecture, motivation, reward and locomotion [1, 2], potentially via ascending and descending, afferent and efferent connections to many brain regions [3]. Studies reported a neuronal loss in the PPN of patients with Parkinson’s disease (PD) [4, 5] and in the most common atypical parkinsonian syndrome, progressive supranuclear palsy [4, 6]. This neuronal loss principally affects cholinergic neurons and may underlie several of the motor abnormalities seen in PD patients. Experimental evidence for this was provided by the selective destruction of the pedunculopontine cholinergic subpopulation in rats [7] and also in macaques [8], through an intra-pedunculopontine infusion of a diphtheria toxin (Dtx) conjugated to the peptide Urotensin II (UII) (Dtx::UII), serving as the endogenous ligand for urotensin-II receptors that are expressed only by cholinergic neurons in this region. Dtx::UII induces a gradual cholinergic-specific cell death by inhibiting the synthesis of choline transporter [9]. Following lesion formation in the rats, no effect was seen on execution of individual motor actions; however, impairments emerged when the demands of the task increased, i.e., during an acrobatic locomotor task (accelerating rotarod) [7]. This result mimicked that reported from the primate study, where the lesion induced no changes in the levels of baseline locomotion; however, the animals displayed significant changes in gait and posture when they were assessed in a guided and trained semi-bipedal walking task [8].

Although no single genetic-based/toxin-induced animal model of PD perfectly recapitulates all neuropathological characteristics and clinical symptoms of the human disorder [10], recent results have highlighted the potential of the lactacystin rat model for studying neuropathological mechanisms underlying PD [11]. In rats receiving a unilateral injection of the irreversible ubiquitin-proteasomal system inhibitor, lactacystin, into the substantia nigra pars compacta (SNc), we recently reported a loss of cholinergic neurons in the nucleus tegmenti pedunculopontine (PPTg), the rodent equivalent of the human PPN [12], which at 5 weeks following the lesion, resembles the level of neuronal loss observed in advanced human PD [11]. The toxin was shown to affect non-cholinergic neurons in this nucleus also, which mimics observations made in post-mortem PPN tissue from PD-affected patients [5, 13]. This includes recent findings [5], which revealed that alpha-synuclein (αSYN) overexpression (resembling Lewy body pathology) affects both cholinergic and non-cholinergic neurons in the PPN of post-mortem PD-affected brains as well as in the PPTg of lactacystin-lesioned rats. In line with this, a loss of both neuronal types was observed in the PD cohort, although *non*-cholinergic neurons were less impacted upon than the cholinergic population [5]. The pathological alterations, concomitant with nigrostriatal deficits and PD-related motor abnormalities seen in the lactacystin rat model of PD [11, 13] strongly supports the notion that a unilateral injection of lactacystin in rats represents a useful experimental tool by which to investigate the impact of PPN cholinergic deficits in PD, over that of more traditional experimental models of PD. This includes 6-hydroxydopamine (6-OHDA) lesioned rats, which failed to induce the PPTg cholinergic cell loss seen in human PD patients [14].

Deep brain stimulation (DBS) of the PPN (PPN-DBS) improves Levodopa-unresponsive postural instability and gait abnormality in PD patients [15–17]. Moro and colleagues [18] presented the first report of the effects of unilateral PPN-DBS in advanced PD cases, which were assessed in a double-blinded manner. The authors reported that patients reported a significant reduction in the frequency of falls during both the ON and OFF stimulation conditions, compared to pre-surgical levels. This improvement was seen at 3 months and persisted until assessment was repeated at 12 months post-surgery. Certain gait parameters showed improvement at 3 months following surgery; however, this effect did not persist until the repeat assessment at 12 months post-surgery. No other major or subjective motor differences were seen during either the ON or OFF stimulation assessment conditions at 3 or 12 months of continuous stimulation. Importantly, the study found that no significant permanent adverse events were induced by the chronic stimulation of the PPN region at 1 year following the surgical procedure. Taken together, although the study suffers from being based on a small cohort (*n* = 6), with a relatively short follow-up period, the study highlighted the potential of PPN-DBS to prevent falls resulting from gait and postural disturbances.

In other recent work, Welter and colleagues [19] conducted a double-blind cross-over, randomized trial of PPN-DBS on PD patients. Although similarly powered as the study by Moro and others [18], the study reported a significant improvement in anticipatory postural adjustments and double-stance duration, but not the length and speed of the first step. Moreover, the intervention alleviated parkinsonian akinesia, while combined PPN-DBS and levodopa treatment produced a significant decrease in freezing episodes. Quality of life also improved significantly with PPN-DBS.

Further support that PPN-DBS could be clinically effective was gained from studies performed on 1-methyl-4-phenyl-1,2,3,6-tetrahydropyridine (MPTP)-lesioned rhesus macaques that showed akinesia [20]. The study found that in these MPTP-induced parkinsonian animals, unilateral low frequency stimulation of the PPN led to significantly increased levels of motor activity, without inducing abnormal involuntary movements as a side-effect to the treatment. In other work by the same group, it was shown that stimulation of the PPN in addition to Levodopa treatment of MPTP-induced parkinsonian monkeys increased animals’ motor activity significantly more than Levodopa treatment alone [21]. However, both the human PPN and the rat PPTg are highly heterogeneous structures, containing cholinergic [22, 23], gamma-aminobutyric acid (GABA)ergic [5, 24], glycinergic [5] and glutamatergic neurons [24]. PPN-DBS stimulates all cell types indiscriminately, leaving it impossible to identify which neuronal subtype underlies clinical improvements. To understand the possible therapeutic effects of stimulating PPTg cholinergic neurons on a range of PD-related motor dysfunction in the lactacystin rat model of PD, we utilised designer receptors exclusively activated by designer drugs (DREADD) [25] to selectively depolarize cholinergic neurons in the PPTg of sham- and toxin-lesioned rats. A viral vector, adeno-associated virus (AAV) containing the floxed muscarinic G protein-coupled receptor, hM3D*q* [26, 27] was stereotaxically infused into the PPTg of choline acetyltransferase (ChAT)::Cre rats, where all cholinergic neurons express Cre-recombinase [28], thus restricting hM3Dq expression to cholinergic neurons. Neurons expressing hM3Dq were stimulated by peripherally administering the synthetic ligand, clozapine-N-oxide (CNO) [26]. An overview of the entire experimental procedures followed is provided in Additional file 1: Figure S1. Effects were examined in four treatment groups: SNc-lactacystin + PPTg-DREADD (L + D), SNc-lactacystin-vehicle + PPTg-DREADD (V + D) rats, contrasting with lesioned and sham-lesioned animals that had received a control viral vector (AAV-channelrhodopsin2 (hChR2)) in the PPTg: SNc-lactacystin-vehicle + PPTg-control virus (V + CV) and SNc-lactacystin + PPTg-control virus (L + CV).

## Results

### Vector-mediated expression of hM3Dq and ChR2 is restricted to Cre expressing ChAT-immunopositive neurons of the PPTg

**Fig. 1:**
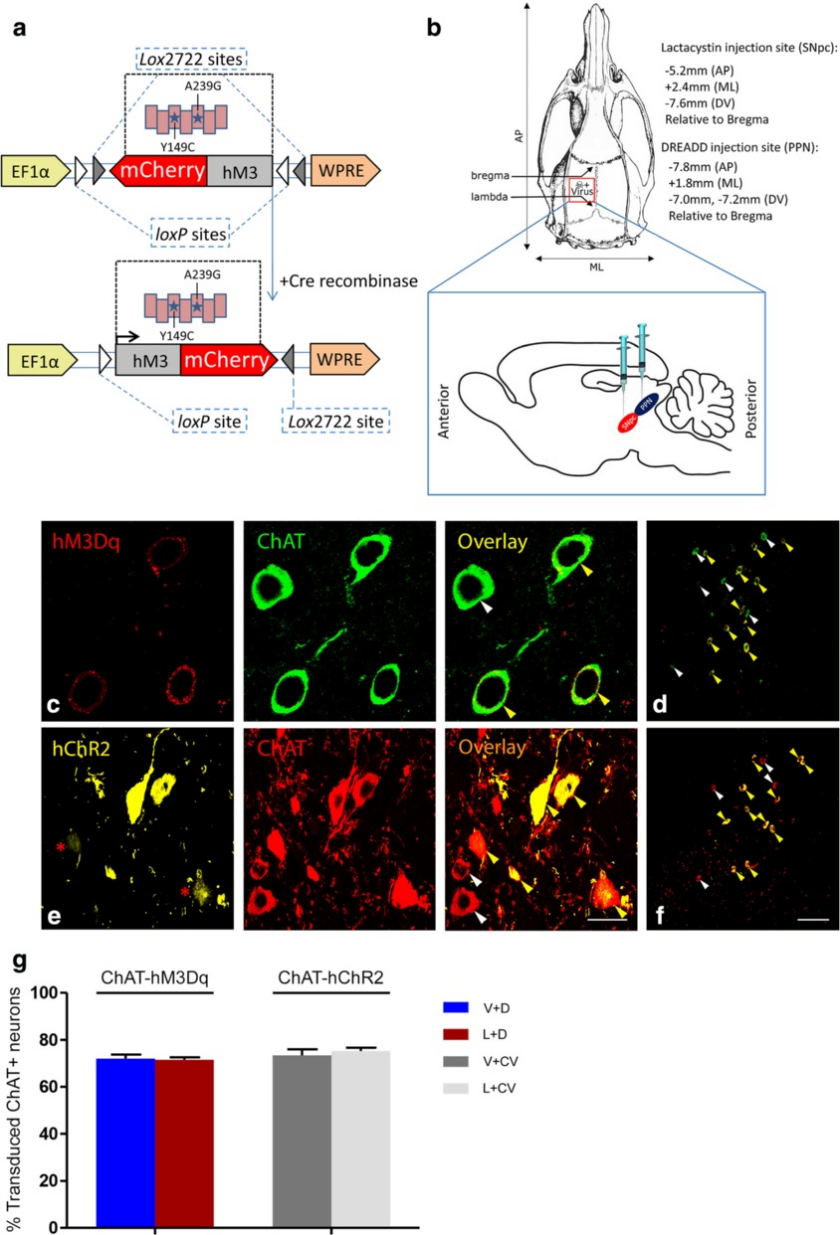
DREADD design and stereotaxic delivery results in high ChAT-DREADD+ neuronal expression within the rat PPTg. **a** Schematic showing the Cre-dependent AAV containing mutant hM3Dq. Upon expression, hM3Dq-mCherry and ChR2-eYFP is inverted to enable transcription from the EF-1a promoter. **b** The location for unilateral stereotaxic injection of either constructs into the PPTg of Chat::Cre-transgenic rats, along with either lactacystin or lactacystin vehicle into the unilateral SNc. **c** High magnification confocal photomicrographs show strong ChAT- immunofluorescence signaling for individual cell bodies that overlap considerably with mCherry-tagged hM3Dq, expressed in the plasma membrane. The intensity of the immunofluorescence signal for ChAT (*green*) significantly surpassed the intrinsic brightness of expressed hM3Dq (*red*), the latter that was induced through viral vector-mediated transduction. Co-expressing neurons appear mainly green, due to the strong immunosignal of the applied secondary antibody for ChAT, but with some yellow labelling. **d** A low magnification image shows the PPTg resident cholinergic neurons (*green*), interspersed with cholinergic neurons co-expressing (*yellow*) mCherry-tagged hM3Dq. **e** High magnification confocal images show red labelled ChAT+ neurons co-expressing (*yellow/orange*) eYFP-tagged hChR2. **f** A low power confocal image shows the high degree of co-expressing ChAT+ (*red*) and yellow labelled hChR2 PPTg neurons. In the case of both hM3Dq and hChR2, viral vector-mediated expression was limited to PPTg ChAT+ neurons. However, some variance in viral vector-mediated expression was seen, especially in the case of ChAT-hChR2, with some individual ChAT+ cells that expressed the gene construct to a lesser extent (visible as a lower fluorescence intensity, such neurons being indicated by a red asterisk in (**e**), left panel) than other transduced cholinergic cells. High power images were taken with a 20×, air-immersion objective lens, while low power images were captured with a 63× oil-immersion objective lens. Arrowheads point to ChAT+ neurons that did (*yellow*) and did not (*white*) express the respective virally transduced constructs. Scale bars: 50 μm (**c**, **e**); 100 μm (**d**, **f**). **g** The degree of overlap between ChAT+ (*green*) and hM3Dq + (*red*) neurons for the V + D (*n* = 12) and L + D rats (*n* = 12) and the overlap between ChAT+ (*red*) and hChR2+ (*yellow*) neurons for V + CV (*n* = 12) and L + CV rats (*n* = 12) revealed that dual expression of the viral vector-ChAT+ PPTg neurons exceeded 70 %, relative to all ChAT+ PPTg neurons. This demonstrates that our approach using Cre-mediated recombination to selectively express DREADD within PPN cholinergic neurons resulted in high transduction efficiency

Stereotaxic administration of AAV-hM3Dq-mCherry (Fig. 1a) into the PPTg of ChAT::Cre rats (Fig. 1b) resulted in the mCherry signal being seen exclusively in the plasma membrane and some axons of PPTg ChAT-immunopositive (ChAT+) neurons (Fig. 1c, d). Similarly, hChR2 administered into the PPTg of ChAT::Cre rats resulted in exclusive detection of yellow fluorescent protein (eYFP), a fluorescent marker for cells expressing ChR2, in ChAT+ PPTg neurons (Fig. 1e, f). hM3Dq-mCherry co-expressed PPTg ChAT+ neurons by >70 % (V + D: 74 ± 2 %, L + D: 72 ± 1 %, *p* = 0.41, NS, Fig. 1g). Similarly, the proportion of neurons co-expressing ChR2-eYFP and ChAT in the PPTg was >70 % in sham and lesioned rats (V + CV: 73 ± 3 %, L + CV: 75 ± 1 %, *p* = 0.67, NS, Fig. 1g). The high level of cholinergic neuronal transduction efficiency seen in all rat groups, confirmed our ability to selectively target the constructs to PPTg cholinergic neuron using this method. Moreover, ChAT+ PPTg neurons co-expressing either hM3Dq-mCherry or hChR2-eYFP, displayed typical cholinergic neuronal morphology, presenting with medium to large somata. The large neurons had 3–6 primary dendrites of a fusiform, triangular or multipolar shape, while medium-sized neurons appeared round or oval, with only 2–3 dendrites, in accordance with previous reports [29, 30]. Furthermore, the particular DREADD made use of here, expressed on the plasma membrane of the transduced target neurons [26]. Taken together, this result indicates that the strategy employed serves as an effective one by which to restrict hM3Dq and ChR2 expression to Cre expressing ChAT+ neurons in the PPTg of rats.

### In rats administered CNO, hM3Dq-, but not ChR2 transduced ChAT+ PPTg neurons show c-Fos immunoreactivity, restricted to the stereotaxically injected hemisphere

**Fig. 2:**
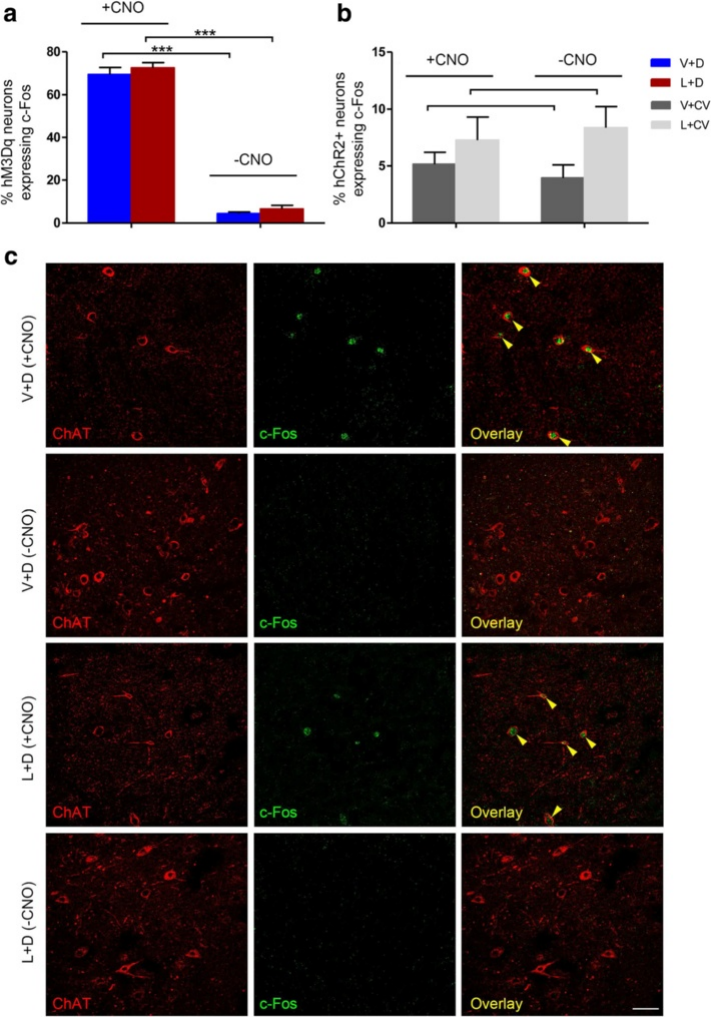
Injection of CNO induces c-fos immunoreactivity in PPTg ChAT+ neurons of rats expressing Cre-dependent AAV-hM3Dq-mCherry. In ChAT::Cre rats where hM3Dq (*red*) was expressed unilaterally in the PPTg and when brains were obtained within 90 min of administering CNO, c-Fos immunoreactivity (green nucleic stain) overlapped with the hM3Dq signal, confirming that CNO stimulates hM3Dq-expressing PPN neurons. **a** c-Fos-expressing cell counts revealed relatively high expression in the ipsilateral but not the contralateral PPTg of V + D and L + D rats (*n* = 12/group). However, c-Fos signalling levels in rats that underwent a CNO wash-out phase lasting ~48 h, prior to sacrifice and brain collection, detected minimal hM3D+ neurons co-expressing c-Fos. **b** In both PPTg-CV rat groups (*n* = 12/group), ChAT-c-Fos neuronal overlap was nearly undetectable, when measured either at + CNO or -CNO. **c** Representative fluorescent microscopy images of contralateral and ipsilateral PPTg c-Fos fluorescence seen in the same brain section but in the different experimental groups. Merged images are shown in the right panels, with yellow indicating co-localisation. Magnification: ×20, scale bar: 25 μm. Mean ± SEM, *p <0.05

The transcription factor c-Fos is an indirect marker of neuronal activity, which upregulates in response to increased neuronal activity [31]. No c-Fos expression was seen in the PPTg in unlesioned hemispheres in any experimental group, thus we restricted analyses to the lesioned (and viral vector infused) side. +CNO resulted in high c-Fos + overlap with hM3Dq + PPTg neurons (V + D: 69.37 ± 3.33 % overlap between ChAT+ and c-Fos + neurons, L + D: 72.52 ± 2.48 % overlap (Fig. 2a). ChAT-c-Fos co-expression was ten-fold lower in hM3Dq-receiving rats when CNO wash-out was allowed prior to brain tissue collection (V + D: 4.35 ± 0.72 % and L + D: 6.53 ± 1.70 %, ***p <0.0001, Fig. 2a).

Conversely, during + CNO, in both PPTg-CV rat groups, % c-Fos-hM3Dq + was very low (V + CV: 5.15 ± 1.06 % and L + CV: 7.3 ± 2.03 %, Fig. 2b). However, during + CNO, the % overlap for V + CV (3.94 ± 1.15 %) and L + CV (8.37 ± 1.87 %) were similar to the ones for V + D –CNO and L + D -CNO (Fig. 2b, c).

### Stereological cell counts validated the expected levels of neuronal loss in the lactacystin-lesioned rats

**Fig. 3:**
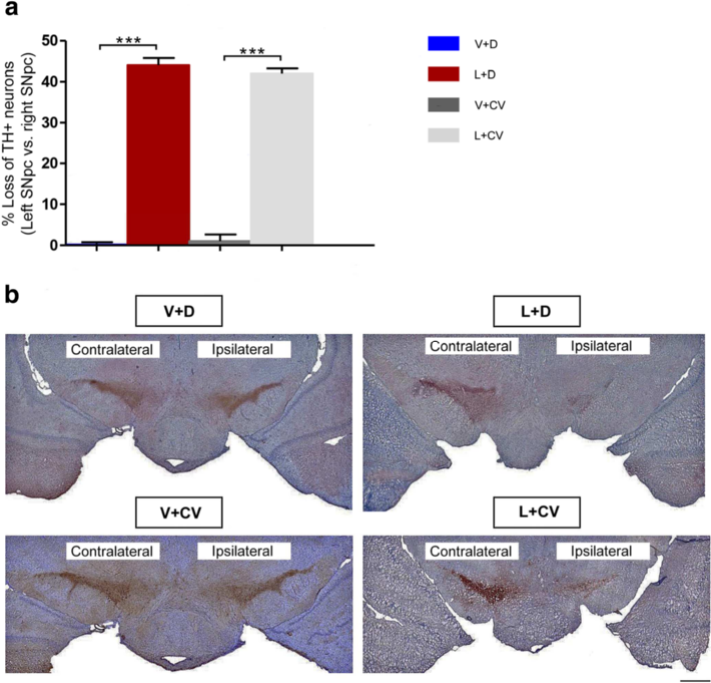
Lactacystin lesioning induces a nigral dopaminergic lesion that closely resembles clinical PD. **a** Stereological quantification of TH+ neurons in the SNc on the lactacystin-lesioned side reveals a near 50 % loss of TH+ neurons, when compared to the same hemispheric side of sham-lesioned rats (*n* = 12/group). Mean ± SEM, ***p <0.001. **b** Representative Bright field images using a 4×/0.10 NA air objective taken of TH+ neurons in coronal brain sections containing the SNc at 5 weeks post-surgery for all experimental groups. Scale bar: 200 μm

Each experimental group (L + D, V + D; L + CV and V + CV; *n* = 12/group) was divided into rats that underwent CNO wash-out (*n* = 6) before brains were removed for analyses and those sacrificed soon (2–3 h) after receiving CNO (*n* = 6), when CNO could still be present. Following unbiased stereological estimation, statistical analyses revealed that systemically-remaining CNO didn’t exert a statistically significant effect on the interhemispheric counts of tyrosine hydroxylase immunopositve (TH+) neurons in any of the rat groups, which prompted us to combine the + CNO and –CNO stereological datasets for each of the experimental groups. The stereologically determined numerical densities of SNpc dopaminergic neurons revealed a substantial loss of TH+ SNpc neurons in lactacystin-lesioned rats (left, injected side: 6,348 ± 442 (L + D); 6,644 ± 624 (L + CV); right, non-injected side: 11,437 ± 334 (L + D); 11,544 ± 446 (L + CV)). On the other hand, stereological cell counts of the total number of SNpc dopaminergic neurons compared favourably between the two hemispheres in the 2 groups of sham-lesioned control rats (left, injected side:

11,515 ± 621 (V + D); 11,462 ± 435 (V + CV); right, non-injected side: 11,537 ± 504 (V + D); 11,622 ± 651 (V + CV)). For the different animal groups, this translated to a mean % differences between the injected left and non-injected right SNpcs of: 44.29 ± 1.78 % (L + D), 0.19 ± 0.57 % (V + D), 42.39 ± 1.21 % (L + CV) and 1.21 ± 1.63 % (V + CV). Specifically, L + D rats’ interhemispheric % cell loss was strikingly increased compared to V + D rats (***p <0.0001, Fig. 3a, b). Erats compared to V + CV ones (***p <0.0001, Fig. 3a, b). A comparison of the interhemispheric cell loss of TH+ neurons between the lactacystin-lesioned (L + D and L + CV) and sham-lesioned control animals (V + D and V + CV) revealed a significant loss of TH+ neurons in the SNpc on the lactacystin-injected brain hemispheric side, compared to the non-lesioned SNpc (***p <0.0001; Fig. 3a, b).

**Fig. 4:**
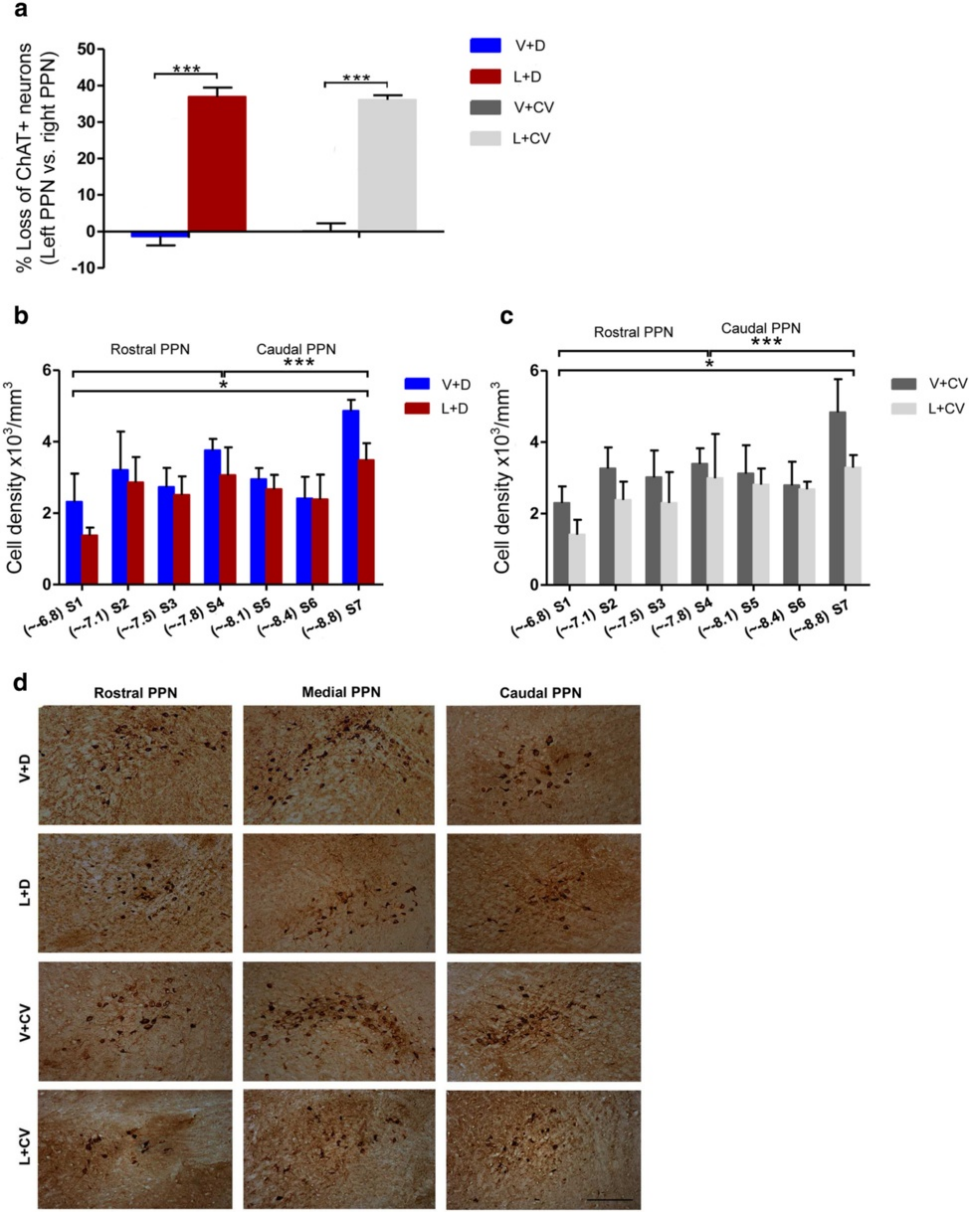
Lactacystin-induced PPTg cholinergic lesions resemble PPN cholinergic neuronal loss seen in post-mortem human PD patients. **a** Stereological cell counts confirmed a decrease in interhemispheric ChAT+ cholinergic neurons of the PPTg in rats that had received an intranigral unihemispheric injection of lactacystin (L + D and L + CV, *n* = 12), compared to sham-lesioned rats (V + D and V + CV, n = 12). V + D vs. L + D rats, ***p <0.001; V + CV vs. L + CV, ***p <0.001. A depiction of cholinergic cell density throughout the rostro-caudal extent of the nucleus reveals that in rats that had been lesioned (L + D and L + CV), cholinergic cell density was decreased throughout the entire rostro-caudal extent of the nucleus, compared to sham-lesioned rats (V + D and V + CV), with neuronal loss noted in even the most caudal parts of the PPTg. Bars depict mean values ± SEM of cholinergic neuronal densities for **b** V + D compared to L + D rats and **c** V + CV compared to L + CV rats. **d** Low-magnification photomicrographs of representative PPTgs in a V + D and L + D rat illustrates how the loss of cholinergic neurons was maintained across the rostro-caudal extent of the PPTg in L + D rats. Scale bar: 200 μm

Data sets were combined for analysing cholinergic stereology data also, as no significant difference was seen between the +0CNO and –CNO data sets in any of the experimental groups. In V + D and V + CV rats, stereological cell counts of the mean total number of PPTg cholinergic neurons, identified as being ChAT+ cells, were similar between the sham-injected SNpc (V + D: 3,108 ± 49; V + CV: 3,097 ± 40) and right, non-injected SNpc (V + D: 3,081 ± 57; V + CV: 3,111 ± 54, *p* = 0.62, NS), translating to a percentage difference of −1.27 ± 2.5 % for V + D rats and 0.42 ± 2.19 % for V + CV ones (Fig. 4a). These data correlate with previous reports of unihemispheric PPTg cholinergic cell counts made in rats [11, 14]. In contrast, lesioned rats (L + D and L + CV) revealed a significant loss of cholinergic neurons on the left, lesioned side (L + D: 1,878 ± 66; L + CV: 1,963 ± 38), compared to the intact opposite hemisphere (L + D: 2,987 ± 26; L + CV: 3,064 ± 28), translating to a loss of 36.94 ± 2.53 % for L + D rats and 35.85 ± 1.41 % for L + CV rats, the interhemispheric neuronal loss for these two groups being marginal (*p* = 0.71, NS, Fig. 4a). Comparison of interhemispheric % cell loss between V + D and L + D animals revealed a significant loss of cholinergic neurons (***p <0.0001, Fig. 4a), validating our previous findings [11]. When comparing V + CV and L + CV rats, the difference was again statistically significant (***p <0.0001, Fig. 4a).

Previous work revealed a higher concentration of cholinergic neurons in the most rostral segments of the PPTg than was seen in the caudal half of the nucleus [23], in non-lesioned rats. Along with data revealing an opposite trend for GABAergic neurons in the rat PPTg [23], this suggests that functional subregions exist within the PPTg. We validated whether a similar cholinergic density distribution was seen in the sham-lesioned ChAT::Cre rats (V + D and V + CV) and whether the PPTg cholinergic density decrease seen in lactacystin-lesioned rats (L + D and L + CV) extended from the most rostral (closest to the SNc, comprising the site of toxin injection) through to the most caudal PPTg sections [11].

Changing densities across the different rostro-caudal segments were similar in all groups (Fig. 4b, c), with the total number of cholinergic cell counts increasing as the distance away from the SNr increased. We followed the accepted means of defining the borders of the PPTg according to the cholinergic neurons; hence the increased density partially reflects the increasing area of the PPTg, as it extends caudally. The main effect of ‘treatment’ on cell density, proved statistically non-significant (*p* = 0.076, NS). However, the other main effect, ‘progressive distance away from the SNc’ (S1-7) on cholinergic cell density, was significant (*p <0.05, Fig. 4b, c) in both groups of lesioned rats, compared to their relative control group. No statistically significant differences were detected. No interaction between the two main effects on cell density was seen. A pair-wise comparison of the mean value for each section, in relation to each rat group was performed which showed no statistically significant differences. Photomicrographs (Fig. 4d) reveal the loss of cholinergic neurons along the rostro-caudal extent of the PPTg.

### During hM3Dq-mediated PPTg cholinergic stimulation, lactacystin-lesioned rats recovered to pre-toxin performance scores obtained in the postural instability test

**Fig. 5:**
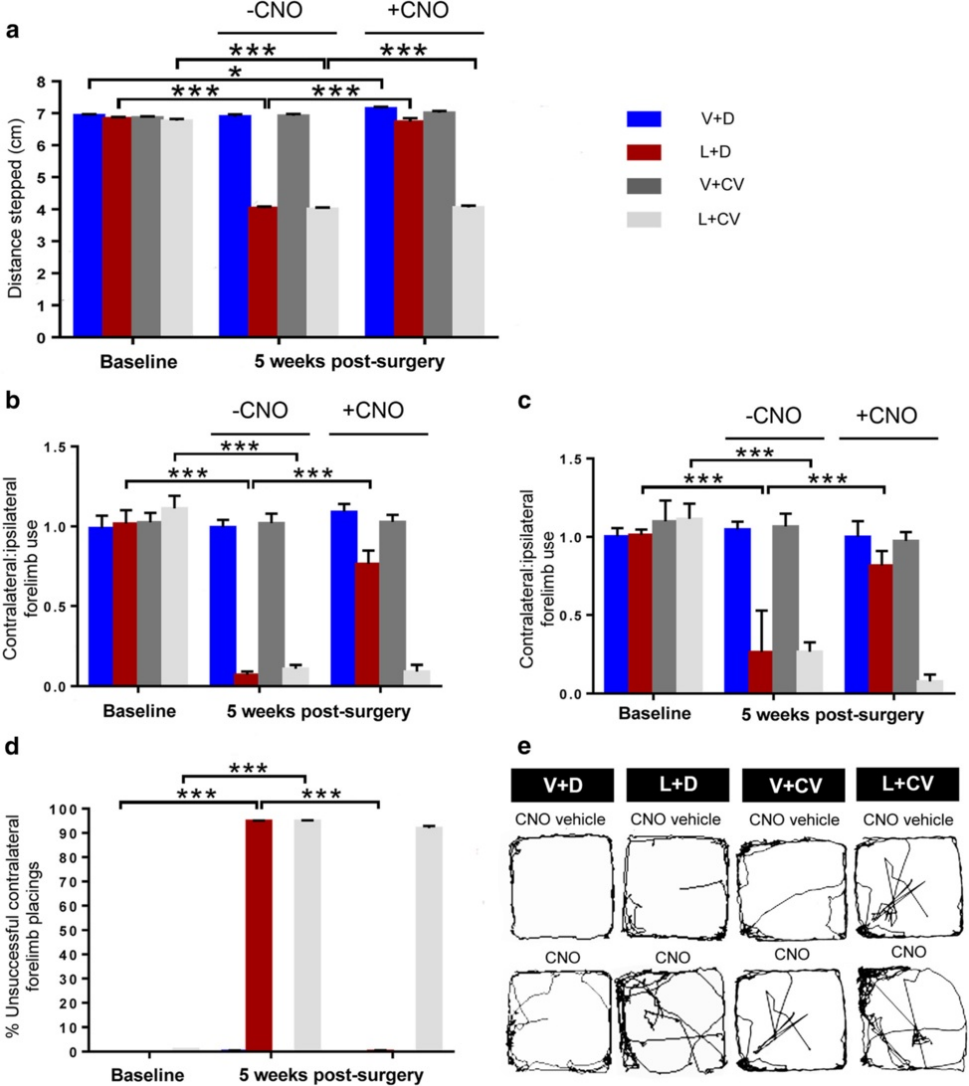
Stimulating PPTg cholinergic neurons drives functional recovery in lesioned rats. **a** Unilateral excitation of PPTg cholinergic improve scores in the PIT in L + D rats (*n* = 12) during + CNO, compared to baseline. A subtle increase in stepping distance by the contralateral forelimb was also seen post CNO in V + D rats (*p = 0.034, *n* = 12). Vertical cylinder test results reveal that PPTg cholinergic-specific stimulation rescued the contralesional limb deficit seen in L + D rats during **b** wall placement and **c** wall "hopping”. **d** Percentage unsuccessful contralateral forelimb placements by rats following stimulation of ipsilateral vibrissae over ten trials. Placement was unsuccessful for nearly all trials in the L + D and L + CV treated rats during -CNO, whereas responses by V + D and V + CV treated rats were near perfect. **e** Representative examples illustrating the distance covered (m) by rats of the different experimental groups showing how, at 5 weeks following the surgery, L + D rats covered significantly less distance in the Open Field arena when measures were taken at -CNO, compared to + CNO. Although the majority of parameters assessed in the Open Field showed complete recovery of function for L + D rats during + CNO in relation to baseline values, the rats of this group did not completely recover to pre-surgical performance levels in terms of ‘distance covered’ (see Table 1). However, a significantly improved effect was seen during + CNO compared to -CNO. In contrast, L + CV rats’ performance decreased between baseline and -CNO, while rats were similarly impaired during + CNO. V + CV rats displayed constant patterns of behaviour throughout all testing phases. Bars depict mean values ± SEM

No significant differences were found for the ipsilateral (non-impaired) forelimb, when considering either the factors ‘treatment group’ or ‘time-point’. Results were then compared between use of the contralateral (impaired) forelimb at baseline and at 5 weeks post-surgery during –CNO vs. +CNO. For V + D rats, there was no significant decline by the contralateral forelimb between baseline and 5 weeks post-surgery during –CNO (*p* = 0.69, NS). However, in this group there was a subtle increase in stepping distance by the contralateral forelimb during + CNO (*p = 0.034), compared to baseline performance (Fig. 5a). For L + D rats, there was a pronounced decline in contralateral forelimb use during –CNO compared to baseline (***p <0.0001). However, during + CNO the stepping distances made by the contralateral forelimb were comparable to that at baseline (*p* = 0.33, NS; Fig. 5a). In V + CV rats, the stepping distances taken by the contralateral forelimb remained consistent between measures taken at baseline and during –CNO (*p* = 0.51, NS) and –CNO vs. +CNO (p *=* 0.42, NS). For L + CV rats, effective use by the contralateral forelimb was dramatically reduced during –CNO, compared to baseline performance (***p <0.0001). In this group, there remained a highly significant impairment in stepping ability by the contralateral forelimb when animals were reassessed during + CNO, compared to baseline performance (***p <0.0001; Fig. 5a). There was no change in contralateral forelimb placement performance from –CNO to + CNO at the 5 week post-surgery testing phase (*p* = 0.67, NS).

### Following CNO administration, significant recovery was also seen in forelimb akinesia in the lactacystin-lesioned rats stereotaxically co-infused with hM3Dq into the PPTg

Use of the contralateral forelimb increased in all domains tested, when comparing + CNO to –CNO stages, but improvement was most evident in wall exploration. The level of “wall placement” by L + D rats showed significantly increased asymmetry between baseline and –CNO (***p <0.0001; Fig. 5b). However, asymmetry scores improved significantly during + CNO, compared to the preceding –CNO stage (***p <0.0001), although the scores did not completely return to baseline level, with a significant difference seen between baseline and + CNO (*p = 0.014). In contrast, V + D rats’ asymmetry scores did not differ significantly between different test stages (Fig. 5b). For L + CV rats, a significant deterioration in symmetry was seen between baseline and –CNO (***p <0.0001), showing no improvement during CNO (*p* = 0.81, NS). Lastly, asymmetry scores remained constant for V + CV rats throughout the different testing conditions, with limb use remaining highly symmetrical (Fig. 5b).

“Serial-stepping” with each forelimb was relatively rare in intact rats, but L + D rats showed increased asymmetry from baseline to –CNO (***p <0.0001; Fig. 5c). During the + CNO condition, symmetry was enhanced significantly (***p <0.0001), compared to –CNO. Performance scores recorded between baseline and + CNO were comparable (*p* = 0.09, NS; Fig. 5c), contrasting with the V + D group, whose performance remained constant throughout all testing stages, similar to that seen for the V + CV rats. However, for L + CV rats, a significant increase in asymmetry (***p <0.0001; Fig. 5c) was seen when comparing baseline to –CNO scores. Performance between –CNO and + CNO continued to decline, although this did not reach statistical significance (*p* = 0.07, NS; Fig. 5c).

### Contralateral forelimb placement showed near complete recovery in the vibrissae-evoked forelimb placement test during hM3Dq-mediated stimulation of PPTg cholinergic neurons in lactacystin-lesioned rats

Correct placements by the ipsilateral forelimb (driven by the non-lesioned cerebral hemisphere) were unaffected by CNO in all of the groups, restricting analysis to the affected contralateral forelimb placements. V + D rats showed no failed responses across any testing sessions (Fig. 5d). L + D rats showed dramatic symptom reversal during + CNO compared to –CNO (***p <0.0001), returning to near-baseline levels (Fig. 5d). V + CV animals performed similarly to V + D rats, while scores obtained by L + CV rats showed severe decline when rats were assessed either at + CNO or –CNO, compared to baseline (Fig. 5d).

### Toxin injected, hM3Dq PPTg stereotaxically infused animals showed a number of significant functional gains post CNO administration in the open field test

Table 1 shows the results of all behavioral parameters analysed during the open field test. Compared to baseline performance, no parameters were affected in V + CV rats during –CNO or + CNO. Although no significant alteration was noted for V + D rats from baseline to –CNO, during + CNO a significant behavioral enhancement was observed for most measures, including distance covered during the trial (Fig. 5e). During –CNO, L + D and L + CV rats revealed a decreased performance in all but one parameter. However, CNO given to L + D rats significantly improved these deficits, with several returning or even exceeding baseline values, whilst CNO failed to reverse any deficits induced by SNc lesioning in L + CV rats. Analyses of the number of ipsilateral rotations displayed by the various groups did not reveal the same clear behavioral profile observed for all other activity measures, with no significant change observed between baseline and –CNO or during + CNO, for any of the experimental groups.

**Table 1:** Open field performance for L + D (a), V + D (b), L + CV (c) and V + CV (d) rats. Hemi-lesioned rats (L + D and L + CV) were severely impaired in all principal motor behaviours and in aspects of complex behaviour, when comparing baseline performance with that at -CNO. When L + D rats were reassessed during + CNO, performance values indicated complete recovery

A	L + D	Baseline	5 weeks post-surgery	
			-CNO	+CNO
Principally motor behavior	Ambulation count (N)	8.92 (±0.54)	5.25 (±0.43)	12.67 (±0.85)
	Average speed (m/s)	0.47 (±0.02)	0.29 (±0.02)	0.48 (±0.02)
	Maximum speed (m/s)	0.74 (±0.02)	0.48 (±0.02)	0.75 (±0.02)
	Time spent mobile (s)	212.13 (±5.16)	193.28 (±2.68)	211.88 (±3.30)
	Distance covered (m)	11.55 (±0.35)	4.66 (±0.39)	6.65 (±0.47
Rotational behavior	Contralateral rotations	4.25 (±0.55)	3.58 (±0.42)	2.75 (±0.28)
	Ipsilateral rotations	3.17 (±0.41)	3.08 (±0.4)	3.08 (±0.48)
Complex behavior	Time spent rearing (s)	51.33 (±2.39)	59.58 (±1.65)	69.58 (±1.72)
	Time spent performing fine motor activity (s)	73.42 (±1.73)	50.50 (±3.61)	73.08 (±2.83)
B	V + D	Baseline	5 weeks post-surgery	
			-CNO	+CNO
Principally motor behavior	Ambulation count (N)	9.25 (±0.59)	8.83 (±0.59)	12.83 (±0.76)
	Average speed (m/s)	0.46 (±0.04)	0.47 (±0.03)	0.48 (±0.02)
	Maximum speed (m/s)	0.72 (±0.3)	0.73 (±0.02)	0.72 (±0.03)
	Time spent mobile (s)	213.40 (±4.5)	211.86 (±3.44)	218.48 (±3.36)
	Distance covered (m)	10.26 (±0.39)	10.17 (±0.32)	11.57 (±0.57)
Rotational behavior	Contralateral rotations	3.50 (±0.39)	1.67 (±0.31)	4.42 (±0.31)
	Ipsilateral rotations	3.25 (±0.41)	3.00 (±0.46)	3.25 (±0.46)
Complex behavior	Time spent rearing (s)	53.67 (±1.67)	54.33 (±3.0)	73.25 (±1.58)
	Time spent performing fine motor activity (s)	75.58 (±2.14)	76.25 (2.48)	78.67 (±2.61)
C	L + CV	Baseline	5 weeks post-surgery	
			-CNO	+CNO
Principally motor behavior	Ambulation count (N)	10.58 (±0.74)	4.83 (±0.39)	4.92 (±0.38)
	Average speed (m/s)	0.51 (±0.04)	0.28 (±0.02)	0.27 (±0.03)
	Maximum speed (m/s)	0.62 (±0.02)	0.45 (±0.02)	0.43 (±0.03)
	Time spent mobile (s)	209.30 (±5.40)	191.70 (±6.06)	190.90 (±5.74)
	Distance covered (m)	11.01 (±0.53)	5.12 (±0.53)	5.05 (±0.51)
Rotational behavior	Contralateral rotations	4.17 (±0.58)	1.58 (±0.42)	1.50 (±0.40)
	Ipsilateral rotations	3.00 (±0.44)	3.25 (±0.39)	3.33 (±0.40)
Complex behavior	Time spent rearing (s)	48.17 (±1.89)	46.42 (±2.08)	45.42 (±1.97)
	Time spent performing fine motor activity (s)	69.00 (±2.48)	47.67 (±4.39)	43.83 (±5.14)
D	V + CV	Baseline	5 weeks post-surgery	
			-CNO	+CNO
Principally motor behavior	Ambulation count (N)	9.67 (±0.66)	9.83 (±0.63)	10.33 (±0.41)
	Average speed (m/s)	0.52 (±0.04)	0.52 (±0.02)	0.52 (±0.02)
	Maximum speed (m/s)	0.69 (±0.04)	1.15 (±0.43)	0.71 (±0.03)
	Time spent mobile (s)	216.17 (±4.31)	217.15 (±3.77)	217.03 (±3.23)
	Distance covered (m)	10.99 (±0.52)	11.07 (±0,43)	11.28 (±0.43)
Rotational behavior	Contralateral rotations	3.50 (±0.59)	3.75 (±0.45)	3.33 (±0.54)
	Ipsilateral rotations	2.83 (±0.47)	3.00 (±0.44)	3.00 (±0.33)
Complex behavior	Time spent rearing (s)	50.83 (±2.09)	48.42 (±1.93)	49.25 (±2.08)
	Time spent performing fine motor activity (s)	77.42 (±1.69)	78.33 (±1.61)	74.83 (±2.29)

### Increased spiking activity of PPTg putative cholinergic neurons seen during CNO-induced activation in hM3Dq-receiving rats and confirmed absence of increased PPTg spike rate activity in rats PPTg-infused with hChR2

**Fig. 6:**
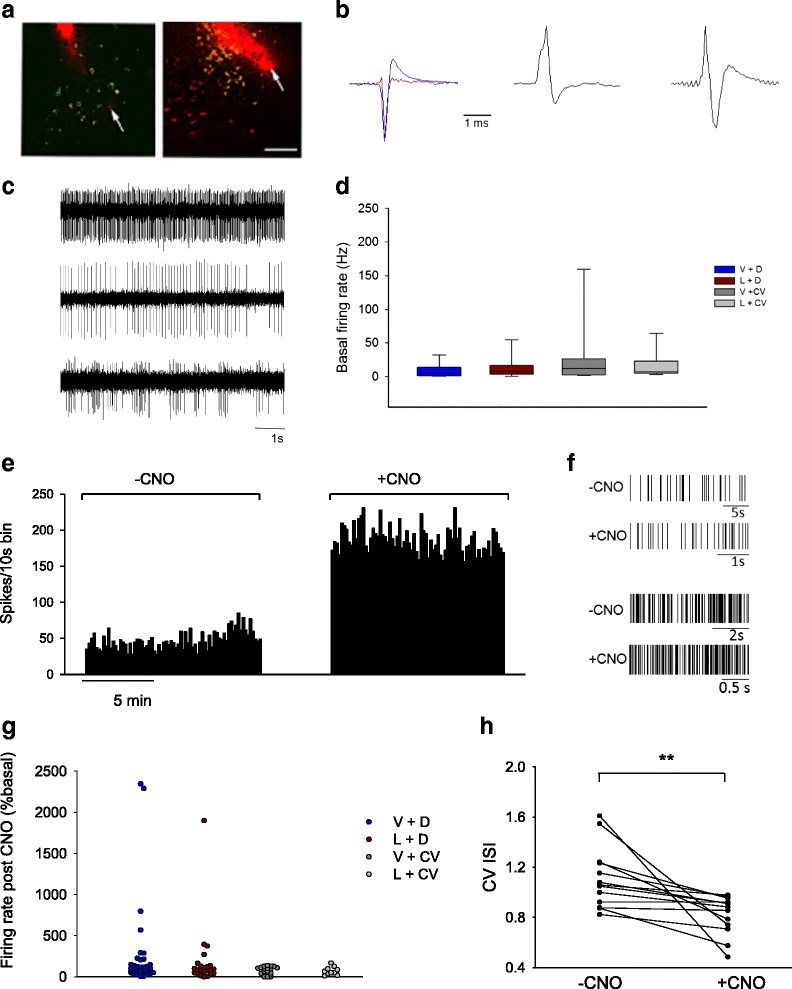
*In vivo* functional assessment of DREADD activity. **a** Correct electrode placement, marked by red DiI for *in vivo* PPTg electrophysiology recordings (a, *top*) shows ChAT+ neurons (green) co-localizing with hM3Dq + ones (*red*). (a, *bottom*) shows ChAT+ neurons (*red*) co-localizing with hChR2+ (*yellow*) ones. Arrows indicate the electrode tips. Scale bar: 20 μm. **b** Average extracellularly recorded action potential waveforms showing example units with predominantly negative deflections (b, *left*) with (*blue*) and without (*red*) a later positive deflection, (b,* centre*) a predominantly positive deflection and (b, *right*) a bipolar positive–negative deflection. Voltage deflections were normalized to the maximum or minimum deflection (100 %). **c** Example traces of neurons firing (c, *top*) regularly, (c, *middle*) less regularly, and (c, *bottom*) phasically, during -CNO. **d** A box and whisker plot of firing rates during -CNO for all groups. Data are median (line), interquartile range (box) and 10th - 90th percentiles (whiskers). **e** Example rate meter histogram depicting a PPTg neuron from an L + D rat, with large increase in firing rate during + CNO compared to -CNO. **f** Example rasters for two neurons responding to + CNO with increased firing rate and clearly increased regularity of firing during + CNO (lower part of each pair) compared to -CNO (upper part of each pair). An expanded time-base during +CNO facilitates comparison of firing patterns. **g** Average firing rates during + CNO, expressed as a percentage of the rate during -CNO for all groups. Each dot represents an individual neuron. **h** CV ISI during + CNO and -CNO for neurons which increased firing rate by >100 %. Differences were highly significant (**p <0.01). Each dot pair (joined by a line) represents an individual neuron

We recorded and analysed a total of 89 putative cholinergic PPTg neurons from 18 animals (1–10 neurons/animal) from all groups. For statistical analysis V + CV and L + CV rats were combined. Examination of coronal PPTg-containing sections revealed that the electrode marked with DiI was correctly placed within the PPTg (Fig. 6a, top and bottom).

The PPTg neurons displayed heterogeneous electrophysiological characteristics. For the majority (72/89) of neurons, the extracellularly recorded action potential displayed a large negative deflection, often followed by a pronounced positive deflection (after hyperpolarization), amounting to approximately a third of the maximal amplitude of the negative deflection (Fig. 6b, top left). In some neurons (13/89) the extracellularly recorded action potential was predominantly a positive deflection, which was generally wider than the negative spikes (Fig. 6b, top right). For the remaining 3/89 neurons the extracellularly recorded action potential was a bipolar positive–negative deflection (Fig. 6b, bottom left). During –CNO, the majority of neurons fired relatively regularly (Fig. 6c, top), a minority of neurons fired irregularly (Fig. 6, middle), while others exhibited distinct phasic activity for at least part of the recording period (Fig. 6, bottom). Firing rates ranged from 0.25–220.27 Hz (Fig. 6d), with the median firing rates (and interquartile range) for the groups that were as follows: V + D, 5.47 Hz (1.50–14.5 Hz); L + D, 7.50 Hz (3.84–16.09); V + CV-L + CV, 7.34 Hz (3.21–21.75 Hz; Fig. 6d). No significant difference was seen between the groups in terms of firing rates during –CNO phase (*p* *=* 0.23, NS). In the V + D and L + D rats, but not V + CV-L + CV rats, +CNO increased the firing rate in a number of neurons above their spike rate during –CNO (Fig. 6e).

In L + D rats, from –CNO to + CNO phases, 4/23 neurons more than doubled their firing rate, whilst 7/23 neurons decreased their firing rate >50 %. The remainder of neurons (12/23) maintained their firing rate within 50-200 % of the –CNO rate. In the V + D group, similar proportions of CNO-responsive neurons were found. Thus 9/38 neurons more than doubled their firing rate during + CNO, while 8/38 neurons decreased their firing rate >50 %. The remainder of neurons maintained their firing rate within 50–200 % of the rate recorded during –CNO. In the combined V + CV and L + CV groups, none of the 28 recorded neurons doubled their firing rate after CNO. In contrast, 11/28 neurons decreased their firing rate by >50 %, while the remaining 17 neurons maintained firing between 50 and 200 % of the –CNO rate.

There was a significant relationship between experimental group and the number of cells that showed increased firing, no change and decreased firing after CNO (*p = 0.042). The most notable difference was between V + D and L + D rats and the combined V + CV-L + CV groups as to number of neurons showing a marked increase in firing during + CNO (4/23, 9/38 and 0/28, respectively).

Table 2 shows the basal characteristics of putative cholinergic PPTg neurons post-CNO. No neurons with very high firing rate during –CNO increased their firing rate after CNO. CNO-responsive neurons were heterogeneous with respect to their basal firing rate and action potential shape. The CNO response (expressed as a percentage of baseline) varied, ranging from 281–1,899 % in L + D rats and 206–2,348 % in V + D rats (Fig. 6f). In all neurons which showed firing rate increases following CNO administration, the increased firing rate was accompanied by increased regularity, defined as decreased CV ISI (*p ≤ 0.05, Fig. 6g, h).

**Table 2:** Putative cholinergic PPTg neurons’ basal firing and action potential waveforms in V + D and L + D rats. Neurons were categorized post-hoc by their responses to CNO, which was defined as ‘increase’ (firing rate during + CNO phase was ≥ 200 % compared to the rate during -CNO phase), ‘no change’ (during + CNO, firing rate was 50–200 % compared to during -CNO) or ‘decrease’ (during + CNO, firing rate was ≤ 50 % compared to during -CNO). The AP shape is classified as positive, meaning that the voltage deflection occurred upwards, negative, meaning that the voltage deflection occurred downwards, or bipolar, where voltage deflections occurred both up- and downwards

Group	Response type	n	AP shape			Basal firing Hz (Median and range)
			Negative	Positive	Bipolar	
V + D	Excitation	9 (24 %)	9	0	0	5.75 Hz (0.3–24.2)
	No response	24 (63 %)	20	3	1	5.473 (0.4–49.3)
	Inhibition	5 (13 %)	4	1	0	3.13 (0.4–31.6)
L + D	Excitation	4 (17 %)	2	2	0	4.09 (0.3–8.8)
	No response	12 (52 %)	11	1	0	10.84 (0.6–83.4)
	Inhibition	7 (30 %)	6	1	0	7.08 (2.3–62.3)

*Abbreviations*: *AP* Action potential

## Discussion

In this study, selective PPTg cholinergic neuronal stimulation utilising expression of an excitatory DREADD which was activated by the selective agonist CNO, resulted in marked reversal of motor deficits in the lactacystin rat model of PD, suggesting that PPTg cholinergic neurons play a vital role in maintaining motor function in dopamine-deficient rats. To confirm that CNO activates PPTg cholinergic neurons, we assessed expression of the immediate early gene, c-Fos, in the PPTg of the rats. c-Fos has been shown to be activated through Gs-mediated signaling events, thus it serves as marker of neuronal activity [32].c-Fos immunoreactity has been used by others as an effective tool by which to validate whether peripheral administration of CNO induces increased intracellular signaling within target neurons locating to restricted parts of the brain, where DREADD had been expressed [33–35]. Approximately two hours after administering CNO, when c-Fos protein levels are elevated [27], large numbers of c-Fos + neurons overlapped with PPTg hM3Dq + ones in both V + D and L + D rats. This contrasted with animals from the same groups that underwent a CNO wash-out phase, which exceeded the nine hours that CNO remains systemically active [27], before animal sacrifice and immunohistochemical processing, where we observed minimal c-Fos expression overlap with PPTg hM3Dq + neurons. Furthermore, CNO failed to induce any c-Fos signal in PPTg hChR2+ neurons in animals stereotaxically injected with the control viral vector (V + CV and L + CV). This confirms that *in vivo* CNO is specific to DREADD receptors, producing transient and alterations of G-protein dependent signaling in the DREADD-transduced target neurons.

*In vivo* electrophysiology revealed that CNO administration increased the firing rate of PPTg putative cholinergic neurons in animals receiving DREADD, but not vector-mediated hChR2 transduced ones. Post-hoc spike data analyses revealed that in all groups, presumed cholinergic neurons excited by CNO were heterogeneous with respect to action potential shape, firing rate and pattern during –CNO. This is consistent with other reports that PPTg cholinergic neurons cannot reliably be distinguished from non-cholinergic neurons by action potential shape, firing rate or -pattern [36].

Previous studies made efforts to classify neurons in the PPTg in terms of their physiological properties. Studies principally using *in vitro* preparations had previously defined four types of PPTg neurons based on their electrophysiological membrane properties [37–39]. Type I neurons were shown to exhibit low-threshold Ca^2+^ spikes (LTS), while Type II neurons displayed a fast transient outward potassium current (A-current). Type II neurons were also shown to display repetitive and rhythmic spontaneous firing with a frequency of 5–15 spikes/s [39]. Type III PPTg neurons are characterized by both LTS and A-current, while Type IV PPTg neurons exhibit neither LTS nor A-current. Reports on the neurochemical phenotype of neurons belonging to these classification criteria vary, with some studies using combined biocytin labelling and ChAT immunohistochemistry showing that 50–60 % of Type 2 and Type 3 neurons are cholinergic, whereas Type 1 and Type 4 neurons were deemed non-cholinergic [37, 39]. On the other hand, Kim and others [40] used combined biocytin labelling and then stained with nicotinamide adenine dinucleotide phosphatediaphorase (NADPH-d), a reliable marker for mesopontine cholinergic neurons [41], with results indicating that 66.7 % of Type 2 neurons and 88.2 % of Type 3 neurons are cholinergic, while the remainder of the Type 2 and Type 3 neurons were non-cholinergic. A small minority of non-cholinergic neurons was classified as being Type IV neurons. No Type I neurons were observed in this study. Therefore, a consistent correlation between the proposed classification system and the neurotransmitter phenotype of recorded PPTg neurons displaying Type I, II, III or IV properties remains to be confirmed, particularly in *in vivo* preparations. Moreover, the classification scheme only allows for distinguishing between cholinergic and non-cholinergic neurons in general, with neurophysiological profiles correlating to a single Type, which were revealed to consist of “mixed” populations, containing both cholinergic and non-cholinergic neurons [37, 42].

A study by Zhang and colleagues [43] found that the majority of presumed cholinergic and non-cholinergic neurons in the unlesioned rat PPTg show a regular firing pattern in normal rats, while in rats that had received an SNc lesion of 6-OHDA, an increased percentage of presumed non-cholinergic neurons exhibited an irregular firing pattern, while the firing pattern of presumed cholinergic neurons remained similar to those recorded in normal rats. Moreover, the study found that lesioning of the nigrostriatal pathway induced an increase in firing rate of both cholinergic and non-cholinergic neurons in the rat PPTg.

We saw a range of firing patterns in CNO-responsive neurons, ranging from irregular and tonic to regular, as well as those showing distinct phasic activity. However, all neurons which showed a large increase in firing rate also showed a decrease in the CV ISI during the + CNO phase. In L + D rats during –CNO, we observed no spontaneous increases in the firing activity of putative cholinergic PPTg neurons. This contrasts with Breit and others [44], who reported that in intranigrally 6-OHDA lesioned rats, PPTg neurons’ spontaneous activity was increased; a result that was subsequently confirmed in studies by Jeon and others [45], hence wise supporting the postulation that the pedunculopontine nucleus is involved in the pathophysiology of parkinsonism. Further interesting findings reported by Breit and others [44] included that ibotenic acid-induced lesioning of the subthalamic nucleus (STN) induced a reduction in the firing rate of the PPTg in normal rats, while normalizing the firing rate in rats that had also received an intranigral 6-OHDA lesion, thereby suggesting that the PPTg is under major control of the STN.

During –CNO, neither action potential shape nor firing rate differed between groups. In V + D and L + D rats, a fifth to a quarter of the recorded neurons showed a large increase in firing rate following CNO administration, contrasting to rats microinfused with the control virus in the PPTg, where no recorded neurons showed a similar response to CNO. Hence we can ascribe the increased firing rate to activation of hM3Dq by CNO. Moreover since our histological data showed h3MDq expression exclusively in ChAT+ neurons, we can presume that the neurons that showed this large excitatory response to CNO to have been cholinergic.

PPTg cholinergic neuronal firing rate became both faster and more regular during + CNO, suggesting that this activity pattern is sufficient for rescuing motor deficits resulting from lactacystin-induced cell loss, synaptic disruptions and circuit disturbances. MacLaren and coleagues [7] demonstrated that the PPTg acts as part of a rapid action selection system, integrating sensory information into motor output. However, in PD there is abnormal processing of sensory information resulting in failure to generate and execute movement [46]. We used the vibrissae**-**evoked forelimb placement test to assess sensorimotor integration across the midline. PPTg cholinergic neuronal stimulation in lesioned rats totally negated failures in forelimb placements following whisker stimulation, but only in L + D and not in L + CV rats. Previous work suggested that this may be due to modulation of the thalamus and cerebral cortex by PPTg cholinergic neurons [47]. Taken together, the c-Fos immunoreactivity and *in vivo* electrophysiology data serve to validate that CNO successfully activated the PPTg target neurons, to provide a plausible neural substrate for motor function recovery seen in the parkinsonian rats.

Posture- or gait disabilities form major deficits in PD patients and contribute to risk of falling, with evidence indicating that PPN cholinergic dysfunction underlies these difficulties [8]. However, in the study performed by Karachi and others [8], only indirect evidence was given that PPN cholinergic neurons underlie these behavioral deficits, as the investigators lesioned the PPN using Dtx::UII, with recent work revealing that the toxin induces PPN GABAergic and glutamatergic neuronal loss in addition to PPN cholinergic neuron loss [48]. The authors noted that the loss of non-cholinergic neurons in the PPN of Dtx::UII lesioned macaques may have been secondary to the primary loss of PPTg cholinergic neurons, but that it is also possible that the Dtx:UII conjugate exerts a degree of toxic action on non-cholinergic neurons, even when it does not bind to the urotensin-II receptor.

Although no single study has given direct evidence that dysfunction affecting the PPN’s cholinergic population underlie postural instability in PD patients, the fact that dopaminergic medication does not fully ameliorate gait dysfunction supports the notion that non-dopaminergic neurons play a prominent contributing role in sustaining gait [49]. In one study, Bohnen and colleagues [50] conducted an *in vivo* positron emission tomography (PET) study and showed that PD patients with a history of falls have significantly decreased thalamic (i.e., PPN) cholinergic innervation compared with PD patients classified as non-fallers, whereas no difference was detected in the degree of nigrostriatal dopaminergic denervation between these two groups. Moreover, Rochester and others [51], using short-latency afferent inhibition as a putative marker of cholinergic function in cortical and subcortical cholinergic systems [52], showed that cholinergic dysfunction plays an important early role in gait abnormalities in PD, and hence represents a potential therapeutic target.

In the current study, the performance of L + D, but not L + CV rats in the PIT was markedly improved during + CNO, supporting a role for PPTg cholinergic neurons in gait control. PPN-DBS for alleviating postural instability and gait disturbances [18] is still regarded as experimental, while studies reported so far provide only indirect evidence for the role of cholinergic neurons in gait and postural control. Our data provide direct evidence that targeting PPN cholinergic neurons alleviates parkinsonian symptoms. A tentative, but speculative explanation for the improved behavioral effects seen in the L + D rats during + CNO may be that PPTg cholinergic neurons exert stimulatory effects on neurons in the PPTg’s output nuclei, such as the SNc and the STN [53–55], with SNc dopaminergic neurons that undergoes substantial degeneration in PD and in animal models of the disease [56], while the STN exhibits hyperactivity following such chronic dopamine loss [57, 58]. The current study lays the foundation for future work to explore whether the behavioral recovery seen here is due to a synaptic interaction between remaining neurons in the PPTg and those in the SNc, or between neurons in the PPTg and those in the STN.

Hemi-lesioned rats display akinesia (poverty or no control over voluntary movements) in the contralesional forelimb during the “cylinder” test [59], the functional deficit correlating with the dopaminergic lesion size [60] and also shows sensitivity to anti-parkinsonian medication [61]. Hence, our finding that PPTg cholinergic stimulation alleviates akinesia in lesioned rats agrees with others who hypothesised that PPN cholinergic deterioration is directly involved in akinesia, as evidenced by the loss of PPN cholinergics in the akinetic syndrome, progressive supranuclear palsy [4, 62].

Our current results also reveal that PPTg cholinergic function appear critical for general motor behavior in an open field enclosure. Such behaviors have a cognitive component and it is therefore not surprising that changes were noted in PPTg stimulated rats, as the PPN holds extensive reciprocal connections with corticostriatal systems with bearing on cognition and emotional reactivity [1, 2].

Future experiments should determine whether any of the motor symptoms shown by intra-nigral lactacystin-lesioned rats show improved metrics, such as a lateralization improvement, in response to the immediate metabolic precursor of dopamine, levodopa and direct dopamine agonists such as apomorphine. In this regard, work by Nakagawa and others [63] revealed that levodopa and apomorphine effectively ameliorated the motor imbalance brought on by a single unihemispheric injection of 6-OHDA into the medial forebrain bundle. Similar experiments using the lactacystin rat model of PD will significantly enhance understanding as to why DBS of the PPN improves levodopa-unresponsive motor symptoms in PD patients [15–17] and whether this effect relies on a neuromodulatory action by the PPN’s remaining cholinergic neurons on the midbrain dopaminergic system.

## Conclusions

Overall, the results presented in this study provide evidence for a cholinergic-based mechanism for a range of motor symptoms, including gait- and postural abnormalities, similar to what is observed in advanced PD. In addition, we demonstrate that transient cholinergic-specific stimulation allows for a striking improvement in motor scores in a realistic rodent model of PD. Therefore, our observations may have an impact on ongoing efforts for optimizing DBS-based therapeutic outcomes in advanced PD patients whilst the minimizing side-effect profile. Taken together, the work serves as a proof-of-principle that similar studies for dissecting out the relative functional contributions made in a cell type-specific manner in brain targets such as the STN [64], deemed promising for alleviating neurological or neuropsychiatric deficits by means of electrical stimulation, can be achieved using DREADD technology, if applied to well-described animal models of neurodegenerative disease.

## Methods

### Drugs

CNO was obtained from Enzo Life Sciences, dissolved in dimethyl sulfoxide (DMSO) and then diluted in 0.9 % saline solution to yield a final DMSO concentration of 10 %, was administered to all animals at a dose of 1 mg/kg. CNO vehicle injections consisted of saline containing 10 % DMSO. D-amphetamine sulphate was purchased from Sigma-Aldrich and administered at a dose of 5 mg/kg, dissolved in 0.9 % saline. All drugs were injected intraperitoneally (i.p.).

### Virus construction

For production of the DREADD, the hM3Dq coding sequence [27] with an mCherry c-terminal tag, was cloned into a FLEX switch cre inducible AAV vector carrying the elongation factor 1 alpha (EF1α) promoter (Fig. 1a). The final vector (1.4 × 10^13^ genomic copies/ml) was sequence verified and then packaged with AAV2 serotype coat proteins by Vector Biolabs (Philadelphia, USA).

An AAV viral construct (7.1 × 10^12^ genomic copies/ml) with floxed inverted ChR2 (containing the H134R mutation), which was fused in-frame with enhanced yellow fluorescent protein (eYFP) and driven by the EF1a promoter, was serotyped with AAV5 coat proteins (Viral Vector Core Facility, UNC, Chapel Hill, USA). Rats infused into the PPTg with this construct controlled for the DREADD virus injection, since hChR2(H134R)-eYFP is activated optogenetically [65], but is unresponsive to CNO. Aliquots of virus lots were stored at −80 °C until use.

### Animals and surgeries

Imperial College London’s ethics review panel approved all experimental protocols. All animal experiments were performed in accordance with the Animals (Scientific Procedures) Act, 1986 (UK). Rats were housed socially (2–3 animals per cage) and maintained on a 12 h light–dark cycle (between 6:30 and 7:00 AM). Access to food pellets and water was provided *ad libitum*.

Long-Evans ChAT::Cre hemizygous rats (Missouri Mutant Mouse Regional Resource Centre, University of Missouri, USA) were bred with Long-Evans wild-type rats (Charles River Laboratories, Germany) to produce Cre-positive offspring. At weaning age, ear punches were collected from each rat from which genomic DNA was extracted to genotype the *Cre recombinase* transgene using a polymerase chain reaction. Only male rats were used in all treatment protocols, as it is currently unknown whether lactacystin lesions in rats induce sexually dimorphic actions, as other neurotoxin-based animal models of PD have been shown to produce [66]. The rats were randomly assigned to one of 4 treatment groups, namely L + D (*n* = 12), V + D (*n* = 12), L + CV (*n* = 12) and V + CV (*n* = 12).

The ChAT::Cre rats were secured in a stereotaxic frame in a flat skull position under anaesthesia (5 % Isoflurane, IsoFlo®, Abbot Laboratories, UK; vaporised into oxygen 1.5 L/min), whilst receiving analgesia (Buprenorphine; Alstoe Animal Health, York, UK). Bupivacaine (Taro, Ireland), locally administered to the scalp, provided local anaesthesia. The skull was exposed and a small burr hole was drilled through the cranium with a dental drill above the left SNc. Lactacystin (4 μl of 2.5 μg/μl, Enzo Life Sciences, UK; 1 μl/min), dissolved in sterile saline (pH 7.4), was delivered using the following coordinates: 5.2 mm anterior-posterior (AP), +2.4 mm medio-lateral (ML) and −7.6 mm dorso-ventral (DV) [12], from bregma, using a 10 μl Hamilton syringe. Sham-lesioned rats received an equal volume of sterile saline only. Saline and lactacystin groups were operated on in a randomised manner, during the same surgical session.

Following the intra-nigral stereotaxic injection, the rats were then infused with the viral construct (1.5 μl) into the left PPTg (coordinates: −7.8 mm (AP), +1.8 mm (ML), −7.0 μm (DV) [11, 13], at rate of 0.2 μl/min. The needle was lowered a further 0.2 μm to deliver an additional 1.5 μl, using a removable 32 gauge needle (7762–05, Hamilton, Reno, USA). All infusions made were driven by a microinjector syringe pump (11 Plus Elite, Harvard Apparatus, Holliston, USA). The needle was left *in situ* for 5 min before slowly retracting from the brain. The skin incision was sutured (removed 7–10 days post-surgery) and rats were left to recover.

### Detailed behavioral procedures

All behavioral assessments were performed by an assessor blind to the treatment condition of the animals. All experimental procedures were conducted during the light portion of the light–dark cycle. Testing was performed at baseline before surgery and repeated at 5 weeks post-surgery, during which behavioral testing commenced 40 min after peripheral administration of CNO-vehicle (−CNO), to compare to the results achieved by the same rats and executing the same testing procedures when CNO (+CNO) was injected into a rat and testing took place 40 min later.

### Amphetamine-induced rotation

To evaluate the extent of the dopaminergic lesion in the SNpc of the rats lesioned with lactacystin, amphetamine-induced ipsiversive rotation was assessed in all lactacystin-lesioned rats (L + D and L + CV), at 3 weeks post-surgery. Following an i.p. injection of the amphetamine solution, rats were individually placed in a large bowl and the net number of rotations made ipsilateral to the lesion was counted over 30 min, beginning at 20 min after the amphetamine injection. Lesioned rats were included in the study if amphetamine induced at least 7 rotations/min ipsilateral rotations.

### Open field

We measured rats’ activities when placed in a white Plexiglass open field arena (90 cm long × 90 cm wide × 60 cm deep). ANY-Maze software (Stoelting ANY-Maze, Wood Dale, USA) quantified parameters from digital camera recordings (5 min/rat) to provide ambulation counts (N), average speed (m/s), maximum speed (m/s), time spent mobile (s) and distance covered (m), contralateral- and ipsilateral rotations and time spent grooming (s).

### Limb-use asymmetry (“cylinder”) test

Unihemispheric administration of lactacystin produces motor asymmetry, allowing comparison of the affected and unaffected limbs. In the limb-use asymmetry (“cylinder”) test, a rat’s use of the ipsilateral (unimpaired), contralateral (impaired) and both limbs for making contact with the inner wall of the cylinder (wall placement) and inner wall exploration (“serial-stepping”) after fully rearing up, was assessed as described elsewhere [11, 59]. For baseline, −CNO and + CNO testing phases, we video recorded 20 rears/rat.

### Postural instability test

The postural instability test (PIT) [59] was performed by holding a rat at 45° in a “wheelbarrow”-like position over a sandpaper-covered surface. The tip of the rat's nose was aligned with the zero line of a ruler. The experimenter restrained one forelimb against the animal's torso while moving the animal forward over the planted forelimb until making a step to regain its centre of gravity. The new position of the nose tip was recorded. Three trials per forelimb were performed per testing session.

### Vibrissae-evoked forelimb placement test

The vibrissae-induced forelimb placement test assesses for sensorimotor integration across the midline [59]. Briefly, the animal was held by the torso and its vibrissae brushed against the edge of a tabletop to elicit a forelimb placing response from the ipsilateral limb. Ten trials per side per test session were performed.

### Animal sacrifice and brain tissue processing

Animals were anaesthetized with sodium pentobarbital (60 mg/kg) before transcardial perfusion with 50 ml heparinized PBS (37 °C), followed by 4 % paraformaldehyde (PFA). Brains were removed and post-fixed in 4 % PFA overnight and then immersed in graded sucrose solutions for ~48 h at 4 °C. Brains were snap-frozen in pre-chilled isopentane, coronally sectioned (30 μm) with a cryostat (Bright Instruments, UK), mounted onto slides (VWR International, UK) and stored at −80 °C until processing.

### hM3Dq- and ChR2 expression

The transduction efficiency of viral constructs within ChAT+ PPTg neurons of ChAT-Cre rats was determined using immunofluorescence and confocal microscopy. Non-specific binding was blocked by incubating with 5 % normal horse serum (Sigma-Aldrich) for 60 min at room temperature (RT) for slides stained for hM3D-ChAT and with 5 % normal donkey serum (Sigma-Aldrich) for ChR2-ChAT. Polyclonal goat anti-ChAT primary antibody (1:100, AB144P, Millipore, Temecula, USA) was applied overnight at 4 °C. For staining the ChR2-ChAT neurons, the sections were washed in PBS, before incubating for 2 h at RT with Alexa Fluor 488-conjugated horse anti-goat IgG for labelling hM3Dq-ChAT neurons and Alexa Fluor 594-conjugated donkey anti-goat IgG secondary antibodies, with both that were purchased from Vector laboratories, Cambridge, UK. Dilutions of the primary and secondary antibodies were made in PBS containing 0.3 % Triton X-100. Sections were washed in PBS, before mounting them with coverslips with ProLong Gold (Invitrogen, Paisley, UK).

Proportions of ChAT+ neurons overlapping with hM3Dq-mCherry + ones and ChAT+ neurons that were also eYFP+ (expressing ChR2) were determined in double immunolabelled, serial cut PPTg sections, spaced 180 μm equidistant apart. Only the injected hemisphere was analysed, using 4 sections/rat. Sections were imaged in a single z-plane with a TCS SP5 II confocal laser scanning microscope (Leica Microsystems, Wetzlar, Germany), fitted with a Leica DFC 320 digital camera and using a 40×/1.4 N.A. oil-immersion objective lens. All ChAT+ cell bodies in areas where the mCherry or eYFP fluorescence could be seen were imaged. An image was taken of each ChAT+ neuron, producing 120–140 images/rat in the V + D and V + CV rats and 30–50 in L + D and L + CV rats. Co-transduction efficiency was determined by analysing the images post-capture using ImageJ v1.45 software (NIH). For ChAT-hM3Dq, the red and green filters were minimized to identify cell bodies that expressed only ChAT (green), mCherry (red) or both. For the ChAT-hChR2 combination, filters were minimized to identify cell bodies expressing only ChAT (red), eYFP (yellow) or both.

### Immunohistochemistry

For immunohistochemical labelling of ChAT+ PPTg neurons and TH+ dopaminergic SNc neurons using 3, 3’-diaminobenzidine (DAB) chromogen, we followed our previously described protocols [11]. Immunohistochemical controls were performed by omitting the primary antibody. Coverslips were applied with DPX mounting medium (Sigma-Aldrich), with sections mounted serially and in a rostro-caudal direction.

### Stereological cell counts

The stereology platform consisted of a computer-based stereology software program (ImagePro, MediaCybernetics, USA) attached to a Nikon Eclipse E800 microscope and a 3CCD camera (JVC Ltd., London, UK). Firstly, the areas of interest (the SNc and PPTg) were delineated under a low magnification view (2.5× air-immersion objective lens). The computer software then created counting frames (200 × 250 μm) that fell within the respective area of interest (AOI). The SNc was delineated from the rest of the brain based on anatomical landmarks and the cytology of SNc neurons, characterised by clear cytoplasmic TH immunoreactivity, with the cells forming a dense population of neurons. The SNc was also distinguished from the other SN subnucleus, the substantia nigra pars reticulata (SNr), as well as the ventral tegmental area (VTA) and the retrorubral area, by referring to published guides on the anatomical landmarks and regional variations in cell density, orientation and morphological boundaries [12, 67].

On the other hand, the PPTg was recognised on the ChAT-stained sections by the PPTg’s characteristic wedge-like shape [11, 14]. Its rostral border consisted of the SNr, with the PPN extending dorso-caudally towards the lateral tip of the superior cerebellar peduncle, at an angle of ~60° from a vertical line [23]. The perimeter of the PPTg was defined as comprising the Ch5 cholinergic neuronal population, regarded as synonymous with the PPTg [22]. We distinguished the PPTg-Ch5 cell group from cholinergic cell group Ch6, comprising the laterodorsal tegmental nucleus, that lies dorsal and caudal to the neighboring PPTg. In this regard, laterodorsal tegmental nucleus was recognised as dorsally abutting the aquaduct, whilst its caudal extreme end is positioned between the locus coeruleus and the fourth ventricle [12, 68].

Stereological estimates of the number of TH+ and ChAT+ neurons were made in the lesioned and non-lesioned brain hemispheres, comparing lactacystin-lesioned rats to control animals (non-lesioned vs. non-lesioned brain hemispheres for each group). Following immunohistological processing of the SNc- and PPTg-containing sections, these were mounted serially, in a rostro-caudal direction. Every 6^th^ section of the series of sections that contained the AOI was analysed using an unbiased stereology approach that makes use of the optical fractionator method, as previously described [69].

Cells were counted under a low power magnification (20× air-immersion objective lens), by a single investigator who was blinded to the animal treatment. The computer software program allowed for automatic and random movement between the sampling areas that included appropriate “acceptance” and “forbidden” lines. The “forbidden line” rule was applied in the counting protocol, which entailed that only neurons that fell within the sampling area or were touching the “acceptance” line were included in the overall count. In addition, a cell was only considered if the bottom of the cell was observed within the volume of the dissector (the height of the section thickness, excluding a 2 μm thick guard zone at the top of the section). The dimensions of the sampling grid were set at 120 × 120 × 5 μm (x, y, and z axes, respectively).

The total area of the counting frame, relative to the area of the AOI gives the area sampling fraction (asf). The height sampling fraction (hsf) was represented by the height of the optical dissector, calculated by taking an average of 3 random points across the section, using a microcator (Hedenhain, Germany), relative to the actual section thickness (30 μm). In addition, the section sampling fraction (ssf) was 1/6, representing every 6^th^ section throughout the AOI. Total cell estimates were then calculated by using the formula: N = n(1/ssf)(1/asf)(1/hsf), where n equals the number of positive cells counted. An estimate of the precision of the stereological estimates derived at, termed the coefficient of error [70], were calculated for all samples used, to yield a value of 0.15 or less, whilst a mean coefficient of error of less than 10 % was obtained for the estimates made within each individual animal.

### c-Fos immunofluorescence

Following the last behavior test, half the number of rats per group were transcardially perfused with 4 % PFA, while the remainder were allowed a CNO wash-out phase (~48 h) before perfusion. To immunofluorescently stain for c-Fos, 10 % normal goat serum was applied to hM3Dq expressing PPTg sections and 10 % normal donkey serum to hChR2 expressing ones, for 60 min at RT. The primary antibody (1:100, anti-c-Fos rabbit polyclonal IgG; SC-52, Santa Cruz Biotechnology, Santa Cruz, USA) was applied overnight (4 °C), followed by secondary antibodies: Alexa Fluor 488-conjugated goat anti-rabbit IgG (1:200, Invitrogen) for c-Fos-hM3Dq and Alexa Fluor 594-conjugated donkey anti-rabbit IgG (1:200, Invitrogen) for c-Fos-ChR2. Antibodies were diluted in Tris-buffered saline containing 0.3 % Triton X-100. Sections were washed in PBS and coverslipped using ProLong Gold mounting medium (Invitrogen). No unwanted background was found in the corresponding immunohistochemical negative controls with the omission of incubation with primary antibodies. Likewise, to ensure that the secondary antibodies did not non-specifically bind to certain cellular compartments, secondary control reactions were performed where the secondary antibody was omitted. This procedure produced no fluorescent signal.

Contiguous, slightly overlapping individual images (×40 magnification, oil, 1.4 NA) of PPTg cholinergic neurons in each hemisphere were taken using a TCS SP5 II confocal laser scanning microscope (Leica Microsystems, Wetzlar, Germany), fitted with a Leica DFC 320 digital camera and stitched together (Image-Pro Plus software v5.1, Media Cybernetics, Bethesda, USA). Image stacks (8 slices separated by 10 μm steps) were gathered from three sections distanced 180 μm apart. Cells were counted in alternate image slices to yield cell numbers for three different positions along the z-axis. The c-Fos, hM3Dq and ChR2 signals were counted separately, after which the individual signals were merged and the number of c-Fos + neurons co-localising with either hM3Dq-mCherry or ChR2-eYFP expressing PPTg cholinergic neurons were counted. Co-localization was calculated by dividing the number of c-Fos–hM3Dq or c-Fos–ChR2 co-positive cells by the number of hM3Dq + or ChR2 + cells, respectively.

### *In vivo* electrophysiology

At five weeks following surgery, *in vivo* PPTg electrophysiological recordings were made. Rats were anesthetized with urethane (1.5–1.8 g/kg) and secured in a stereotaxic frame. The skull was exposed and a hole was drilled over the left PPTg using the same coordinates as was used for delivering the DREADD or control virus. A silicon electrode array with 16 iridium contacts, spaced at 100 μm and in a linear formation (A1x16-10 mm-100-177-A16, NeuroNexus Technologies, Ann Arbor, USA) was then implanted perpendicularly to the dorsal surface of the rat’s skull. Using a fine control hand-driven micromanipulator, the tip of the recording electrode was positioned at the most dorsal border of the PPTg [12] and slowly advanced until a recording position was found where a number of channels (a total of 16 was available) showed spike discharges. The probe was never descended to a depth exceeding the reference coordinates given for the PPTg’s most ventral edge (−7.8 mm), at this AV-ML coordinate [12]. The probe then remained in position throughout the two recording stages. This consisted of an i.p. administration of CNO vehicle. At 40 min post-injection, the neurons were recorded for 12 min. This was followed by an i.p. injection of the CNO solution. Again, neuronal activity was recorded for 12 min, also at 40 min post-injection. Both CNO vehicle and the CNO solution were administered at a dose of 1 mg/kg, similar to that given for the various behavioral assessments.

Extracellular signals were amplified (×1000) and band-pass filtered (150 Hz–9 kHz), using a PBX preamplifier (Plexon, Dallas, USA), passed through Humbug filters (Digitimer Research Instruments, Hertfordshire, UK) and digitized (Micro 1401–3, Cambridge Electronic Design, Cambridge, UK) at a sampling rate of either 16.7 or 20 kHz. Spiking activity was recorded and captured on a PC using Spike2 software (v7.10b, Cambridge Electronic Design). Following the recording session, the rat was euthanized, perfused and the brain removed. To histologically verify the recording site, the probe was painted with red lipophilic fluorescent dye, DiI (Invitrogen). Only the data of rats where immunofluorescent images showed that the electrode had traversed the PPTg successfully was used in the analyses of the electrophysiological recording data.

Spike sorting was performed manually using Offline Sorter software (Plexon, Dallas, USA). For each recorded channel, thresholds for spike detection were set manually. Waveforms passing the threshold were distinguished using principal component analysis. Consistent and distinguishable waveforms were considered to be extracellularly recorded action potentials from single units. For all well-isolated units which fired spontaneously before administering CNO, time stamp data were exported to NeuroExplorer4 (Nex Technologies, Madison, USA). Firing rate and the co-efficient of variation of the interspike interval (CV ISI) was determined during two 12 min periods: −CNO (commencing 40 min after administering CNO vehicle) and + CNO (commencing 40 min after administering CNO).

### Statistical analyses

The vibrissae-evoked forelimb placement test data was analysed using a Chi-square test. A two-way ANOVA, followed by Tukey post-hoc testing compared ipsilateral to contralateral hemispheric SNc TH+ and PPTg ChAT+ cell loss in lesioned vs. sham-lesioned rats. A mixed design (or split-plot) ANOVA was used for the PIT and cylinder test. For analyzing the *in vivo* electrophysiology data sets, firing rates of recorded PPTg neurons were analysed with a Kruskal–Wallis test. The proportion of cells showing excitatory responses in the four groups was assessed using contingency table analysis (the Monte Carlo simulation option). c-Fos expression data was analyzed using a one-way ANOVA, followed by a Tukey post-hoc test. Data was analysed using Statistical Package for Social Sciences software (v19, IBM). *P*-values were designated as: ***p <0.001, extremely significant; **p ≤ 0.01, highly significant; *p ≤ 0.05, significant and *p* > 0.05, non-significant (NS). Graphical data is expressed as the means ± standard error of the mean (SEM) or median and interquartile range.

## Additional file

Additional file 1: Figure S1. A schematic showing the sequential protocol followed for the *in vivo* experiments conducted on the rats during the +CNO and –CNO phases. (TIFF 2395 kb)

## Abbreviations

AAV: Adeno-associated virus
αSYN: Alpha-synuclein
AP: Anterior-posterior
ChR2: Channelrhodopsin
ChAT: Choline acetyltransferase
CV: Coefficient of variation
CV ISI: Co-efficient of variation of the interspike interval
DBS: Deep brain stimulation
DREADD: Designer receptor exclusively activated by designer drug
DAB: 3, 3′-diaminobenzidine
DMSO: Dimethyl sulfoxide
DV: Dorso-ventral
eYFP: Enhanced yellow fluorescent protein
EF1α: Elongation factor 1 alpha
GABA: Gamma-aminobutyric acid
i.p.: Intraperitoneally
LTS: Low-threshold spike
ML: Medio-lateral
PPTg: Nucleus tegmenti pedunculopontini
NS: Non-significant
NA: Numerical aperture
PPTg: Nucleus tegmenti pedunculopontine
PFA: Paraformaldehyde
PD: Parkinson’s disease
PPN: Pedunculopontine nucleus
PET: Positron emission tomography
PIT: Postural instability test
RT: Room temperature
L+CV: SNc-lactacystin+PPTg-control virus
L+D: SNc-lactacystin+PPTg-DREADD
V+D: SNc-lactacystin-vehicle+PPTg-DREADD
V+CV: SNc-lactacystin-vehicle+PPTg-control virus
6-OHDA: 6-hydroxydopamine
SEM: Standard error of the mean
SNc: Substantia nigra pars compacta
SNr: Substantia nigra pars reticulata
STN: Subthalamic nucleus
TH: Tyrosine hydroxylase
VTA: Ventral tegmental area
