# Supplementary figures and images for: Pharmacogenetic stimulation of cholinergic pedunculopontine neurons reverses motor deficits in a rat model of Parkinson’s disease
Source: Mol Neurodegener. 2015 Sep 23;10:47. doi: 10.1186/s13024-015-0044-5 (PMC4580350; doi:10.1186/s13024-015-0044-5)

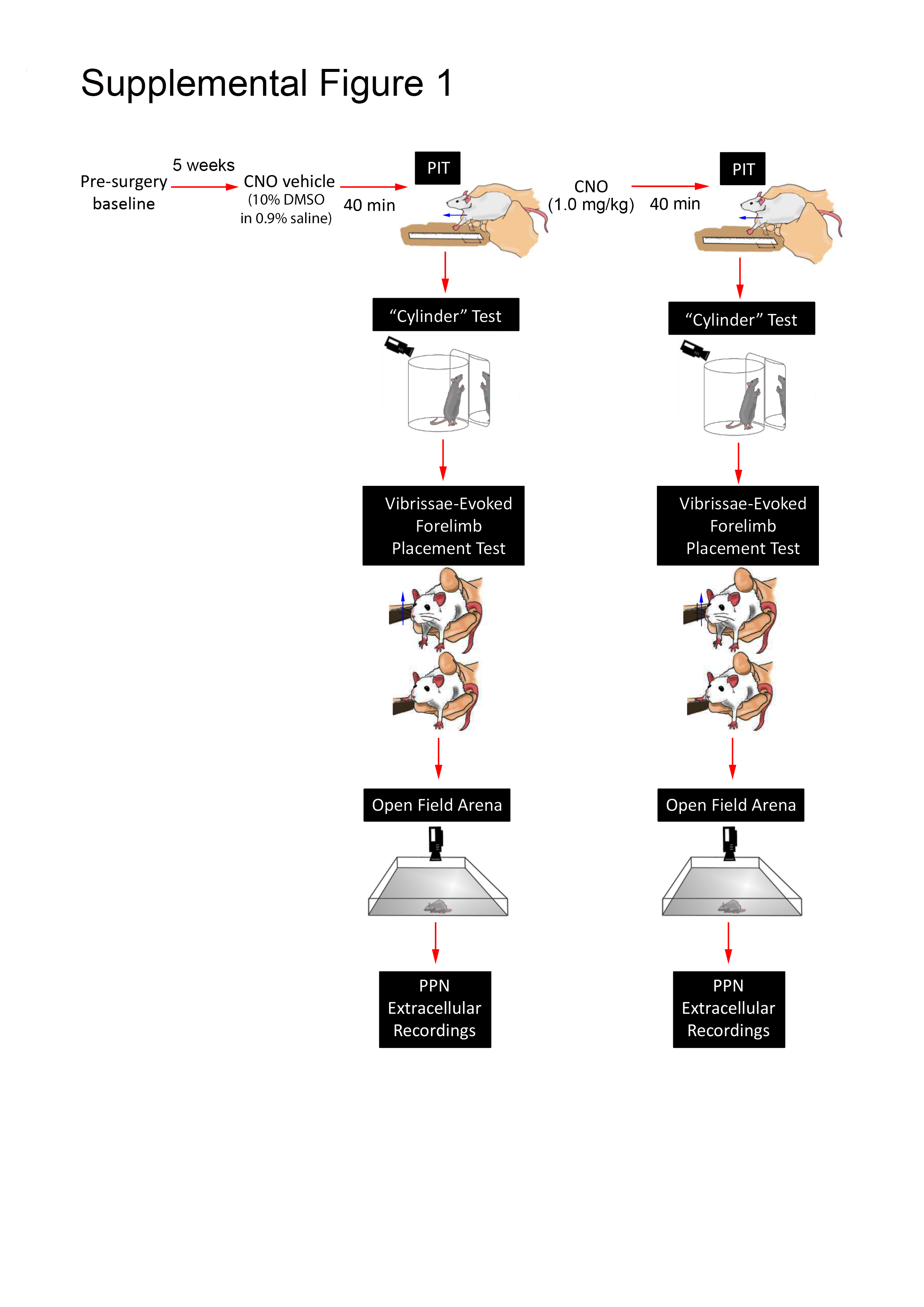

Supplement: Additional file 1: Figure S1. — A schematic showing the sequential protocol followed for the in vivo experiments conducted on the rats during the +CNO and –CNO phases. (TIFF 2395 kb) [file 13024_2015_44_MOESM1_ESM.tif]
